# A redox probe screens *MTHFD1* as a determinant of gemcitabine chemoresistance in cholangiocarcinoma

**DOI:** 10.1038/s41420-021-00476-2

**Published:** 2021-05-01

**Authors:** Ruogu Pan, Zhiqing Yuan, Yingbin Liu, Xuxu Sun, Guiyang Wang, Xiaopen Wang, Junwen Qu, Jian Wang, Jie Yang, Yuzheng Zhao, Yi Yang, Kewei Li

**Affiliations:** 1 Department of Biliary and Pancreatic Surgery, Renji Hospital, School of Medicine, Shanghai Jiaotong University, Shanghai, 200127 China; 2 Department of Biochemistry and Molecular Cell Biology, State Key Laboratory of Oncogenes and Related Genes, Shanghai Key Laboratory forTumor Microenvironment and Inflammation, Shanghai Jiaotong University School of Medicine, Shanghai, 200025 China; 3 Optogenetics & Synthetic Biology Interdisciplinary Research Center, State Key Laboratory of Bioreactor Engineering, Shanghai Collaborative Innovation Center for Biomanufacturing Technology, East China University of Science and Technology, Shanghai, China; 4 Shanghai Key Laboratory of New Drug Design, School of Pharmacy, East China University of Science and Technology, Shanghai, China; 5 CAS Center for Excellence in Brain Science, Shanghai Institutes for Biological Sciences, Chinese Academy of Sciences, Shanghai, China

## Abstract

Cholangiocarcinoma (CCA) is a type of solid tumor derived from the bile duct epithelium that features universal gemcitabine resistance. Here, we utilized a gene-encoded ROS biosensor probe (HyPer3 probe) to sort subpopulations with different redox statuses from CCA cells. The isolated HyPer-low subpopulation CCA cells, which exhibited relatively lower cellular ROS levels, exhibited higher chemoresistance to gemcitabine than HyPer-high subpopulation CCA cells in vitro and in vivo. Mechanistically, increased expression of *MTHFD1* was found in HyPer-low cells. Knocking down *MTHFD1* in HyPer-low cells enhanced cellular ROS and restored sensitivity to gemcitabine. Furthermore, the *MTHFD1* inhibitor antifolate compound methotrexate (MTX) increased cellular ROS, and combining gemcitabine with MTX effectively suppressed cholangiocarcinoma cell growth. In summary, the *MTHFD1* level mediated the heterogeneous cellular redox status in CCA, which resulted in chemoresistance to gemcitabine. Our data suggest a novel strategy for CCA chemotherapy.

## Introduction

Cholangiocarcinoma (CCA), a molecularly heterogeneous neoplasm that arises from the epithelial cells of the bile duct, is one of the most prevalent digestive system neoplasms in adults with high mortality and poor prognosis^1,2^. The incidence and mortality rates of CCA have increased globally, especially in northeastern Asia. Data from the National Cancer Institute show that the 5-year survival rate of CCA is ~8–10%^3^. Most patients are at the advanced or metastatic stage and have already lost surgical indications^4^. For these patients, chemotherapy is the major nonsurgical approach^5,6^.

Gemcitabine was used to treat various solid malignant tumors, including CCA, due to its nucleoside analog deoxycytidine (2’,2’-difluoro 2’-deoxycytidine) property^7^. The regimen that combines gemcitabine with cisplatin or other reagents is the first-line treatment for patients with locally advanced and metastatic bile duct tumors, but poor sensitivity to gemcitabine is commonly observed among them^5,6^. Along with an increasing number of signaling pathways and several targets, such as PI3K/Akt, Erk, and NF-κB, identified in gemcitabine resistance in CCA cells, the determining mechanism needs to be uncovered^8^.

Notably, it has been reported that reactive oxygen species (ROS) can confer sensitivity to gemcitabine to pancreatic cancer cells and gallbladder cancer cells^9,10^, and it is well known that the cellular redox status is correlated with the chemoresistance of various solid tumors, such as HCC^11^, lung cancer^12^, esophageal cancer, stomach cancer, and cervical cancer^13–15^. However, the relationship between ROS and CCA chemoresistance is still unclear.

The cellular redox status maintaining dynamic equilibrium under the physical state results from ROS production and elimination systems^16^. The generation system simply consists of two categories: biological processes such as mitochondrial oxidative metabolism and signal transduction processes such as a cellular response to cytokines and xenobiotics^17^. Compared to the simple ROS generation system, the ROS elimination system operates in an inverse way and consists of two main ways^17^. One of these is to directly eliminate ROS and includes superoxide dismutases (SOD), glutathione peroxidase (GPx), glutathione S-transferase pi (GST-pi), metallothionein-3 (MT3), ferritin heavy chain (FHC), and dihydrodiol dehydrogenase (DDH1 or AKR1C1)^18,19^. Meanwhile, the generation of NADP/NADPH, such as the pentose phosphate pathway and methylenetetrahydrofolate dehydrogenase (MTHFD1), also contributes to the balance of the cellular redox status^20,21^. The long-term unsolved questions are what is the heterogeneous cellular redox status in various cancer cells and how do intrinsic ROS affect the constitutive chemoresistance.

To uncover the correlation between the intrinsic cellular redox status and CCA gemcitabine chemoresistance, we applied a novel tool, the HyPer3 probe, to sort out cellular redox status differences in a subpopulation of CCA cells and found that HyPer-low CCA cells exhibited higher gemcitabine chemoresistance than others with higher *MTHFD1* expression. Since *MTHFD1* is associated with NADPH production and the enzyme providing one-carbon unit derivatives of tetrahydrofolate, the key role and mechanism of the cellular redox status and *MTHFD1* in CCA cell chemoresistance to gemcitabine is worth noting.

## Materials and methods

### Cell culture and reagents

The cholangiocarcinoma cell line QBC939 and HUCCT1 were purchased from the Cell Bank of the Chinese Academy of Sciences (Shanghai, China). The human embryonic kidney 293T (HEK293T) cells were purchased from the American Type Culture Collection. QBC939 and HUCCT1 were all MTHFD1 WT genotype (MTHFD1^+/+^). QBC939, HUCCT1, and HEK293T cells were cultured in Dulbecco’s modified Eagle’s medium (Gibco). All cell lines were supplemented with 10% fetal bovine serum (Gibco), penicillin (100 mg/ml) and streptomycin (100 mg/ml) and were incubated in a humidified chamber with 5% CO_2_ at 37 °C. Gemcitabine, Methotraxate, and puromycin were purchased from MedChemExpress (MedChemExpress, Monmouth Junction, NJ).

### Plasmids and siRNA and

The ORF sequences of HyPer3 were cloned into the pCDH vector with puromycin in the C-terminus. MTHFD1-targeting siRNA and non-specific control siRNA (siNC) used in this study were obtained from Biochemistry and Molecular Cell Biology, Shanghai Jiaotong University School of Medicine.

### Cell apoptosis assays

QBC939 and HUCCT1 cells were plated in dishes or microplates, overnight and treated with gemcitabine or vehicle for 48 h under 1 and 10 μM. After that, all cells were collected by trypsinization without EDTA, and 2 × 10^6^ cells were doubly stained with annexin-V-APC/PI (BD Bioscience) and analyzed by fluorescence-activated cell sorting analysis.

### Experimental animal model

For subcutaneous injection models, cells were resuspended in 50 μL phosphate-buffered (PBS) and mixed with 50 μL Matrigel (BD Biosciences, Billerica, MA), then the cell mixture was implanted subcutaneously into the both flank of the 6-week-old mice (male BALB/c nude mice). Tumors were measured every two days and calculated by the following formula: Volume = 0.5 × Width^2^ × Length, with four mice per group. The tumor volume and weight presented as the means ± S.D (*n* = 3–4). All procedures involving animals were approved and performed in accordance with the Animal Care and Use Committee of Shanghai Jiaotong University.

### Chemoresistance assay

Chemotherapy-induced cytotoxicity was assayed by the Cell-Counting Kit-8 (MedChemExpress, Monmouth Junction, NJ). Briefly, cells were plated in 96-well plates at 5 × 10^3^ cells/well to attach overnight, then treated with Gemcitabine (Gem; MedChemExpress, Monmouth Junction, NJ) at various concentrations. The cell-counting kit-8 assay was performed according to protocols from manufacturer. All experiments were performed independently at least 3 times, and each experiments contained triplicates.

For in vivo chemoresistance nude mice model, 5 × 10^6^ QBC939 HyPer-low cells or 5 × 10^6^ QBC939 HyPer-high cells were resuspended in 50 μL PBS and mixed with 50 μL Matrigel, then implanted subcutaneously into the left flank of 6-week-old mice (male BALB/c nude mice). After 1 week, the mice were intraperitoneally injected with PBS or gemcitabine (50 mg/Kg body weight) twice per week for two weeks. Tumor growth was measured every two days and calculated by the following formulation: Volume = 0.5 × Width^2^ × Length, with four mice per group. The tumor volume and weight presented as the means ± S.D (*n* = 3–4).

For in vivo anti-chemoresistance nude mice model, 5 × 10^6^ QBC939 HyPer-low cells or 5 × 10^6^ QBC939 HyPer-high cells were resuspended in 50 μL PBS and mixed with 50 μL Matrigel, then implanted subcutaneously into the left flank of 6-week-old mice (male BALB/c nude mice). After 1 week, the mice were intraperitoneally injected with gemcitabine (50 mg/Kg body weight) twice per week for two weeks. Tumor growth was measured every two days and calculated by the following formulation: Volume = 0.5 × Width^2^ × Length, with four mice per group. The tumor volume and weight presented as the means ± S.D (*n* = 3–4).

### Flow cytometric analysis

The cell were transfected with HyPer3 probe plasmid 48 h before. The cells were treated with H_2_O_2_ or DTT and analyzed using a flow cytometer (BD, Biosciences). The cells treated with PBS was served as a control. For cell sorting, its based on the ratio of F488nm/F405nm, followed by sorting with a flow cytometer (BD, Biosciences).

### Immunofluorescence staining

Tissues were seeded on the cover slides and fixed in 4% paraformaldehyde over night and treated with 0.05% Triton X-100. Then the tissue were blocked for 30 min in 10% bovine serum albumin (BSA; Sigma-Aldrich) in PBS and then incubated with fluorescence-conjugated secondary antibodies at room temperature for 1 h. DAPI (4,6-diamidino-2-phenylindole) was used to stain nuclei. Immunofluorescence signals were captured by a fluorescence microscope (Leica Biosystems, Nubloch, Germany).

### Western-blot assays

Immunoblotting was performed using standard procedures. Cell lysates were prepared in radioimmunoprecipitation lysis buffer (50 mM Tris, pH 7.4, 150 mM NaCl, 1% NP-40, 0.1% sodium dodecyl sulfate (SDS), 2 μM EDTA) containing proteinase inhibitor and were quantified with the Micro BCA Protein Assay Kit (Thermo Fisher Scientific). Aliquots of 20 μg protein were electrophoresed through 10% or 15% SDS polyacrylamide gels and were then transferred to polyvinyl difluoride membranes (Millipore), followed by blocking in 5% skim milk at room temperature for 1 h and incubated with primary antibodies at 4 °C overnight. Secondary antibodies were labeled with horseradish peroxidase, and the signals were detected using the ECL Kit (Millipore). The images were analyzed using ImageJ 1.43 software. β-Actin served as an internal control for the whole-cell lysates. Antibody against MTHFD1 (A8661, dilution 1:1000) was from Abclonal; β-Actin (A1978, dilution 1:1000) was from Sigma-Aldich.

### RNA extraction and real-time quantitative PCR (RT-qPCR)

Total RNA was extracted from cells using Trizol Reagent (Life technology) following the manufacturer’s protocol, and 1 μg of total RNA was reverse transcribed using the PrimerScript RT Reagent Kit (TaKaRa) into cDNA. RT-qPCR was performed in Roche Light Cycler 480 Real-Time PCR system (Roche). The Ct values obtained from different samples were compared using 2^−△△Ct^ method, and β-Actin or GAPDH served as an internal reference gene.

### NADPH/NADP^+^ assay

Cellular ratio of NADPH/NADP^+^ was assayed by the NADP/NADPH Assay Kit (Abcam). Briefly, sorted cells were plated in 96-well plates at 5 × 10^3^ cells/well to attach overnight. The NADP/NADPH Assay Kit was performed according to protocols from manufacturer. All experiments were performed independently at least 3 times, and each experiment contained triplicates.

### Statistical analysis

Data were presented at the mean ± S.D. one-sample Kolmogorov–Smirnov test was applied for normally distributed data examination. For normal distribution data, two tailed-student’s *t* test was applied to compare the difference between two groups, and one-way analysis of variance test was applied to compare the difference among three or more groups; for non-parametric data, Mann–Whitney *U* test (data with abnormal distributions) was applied. For survival analysis, the Kaplan–Meier method and log-rank test were applied to determine the OS. Fisher’s exact tests were applied to analyze the correlation. All statistical calculation was performed using SPSS software package (version 23.0, IBM SPSS), and a *P* < 0.05 was considered to be statistically significant.

## Results

### The redox status was heterogeneous in cholangiocarcinoma, which was revealed by HyPer3

**Fig. 1:**
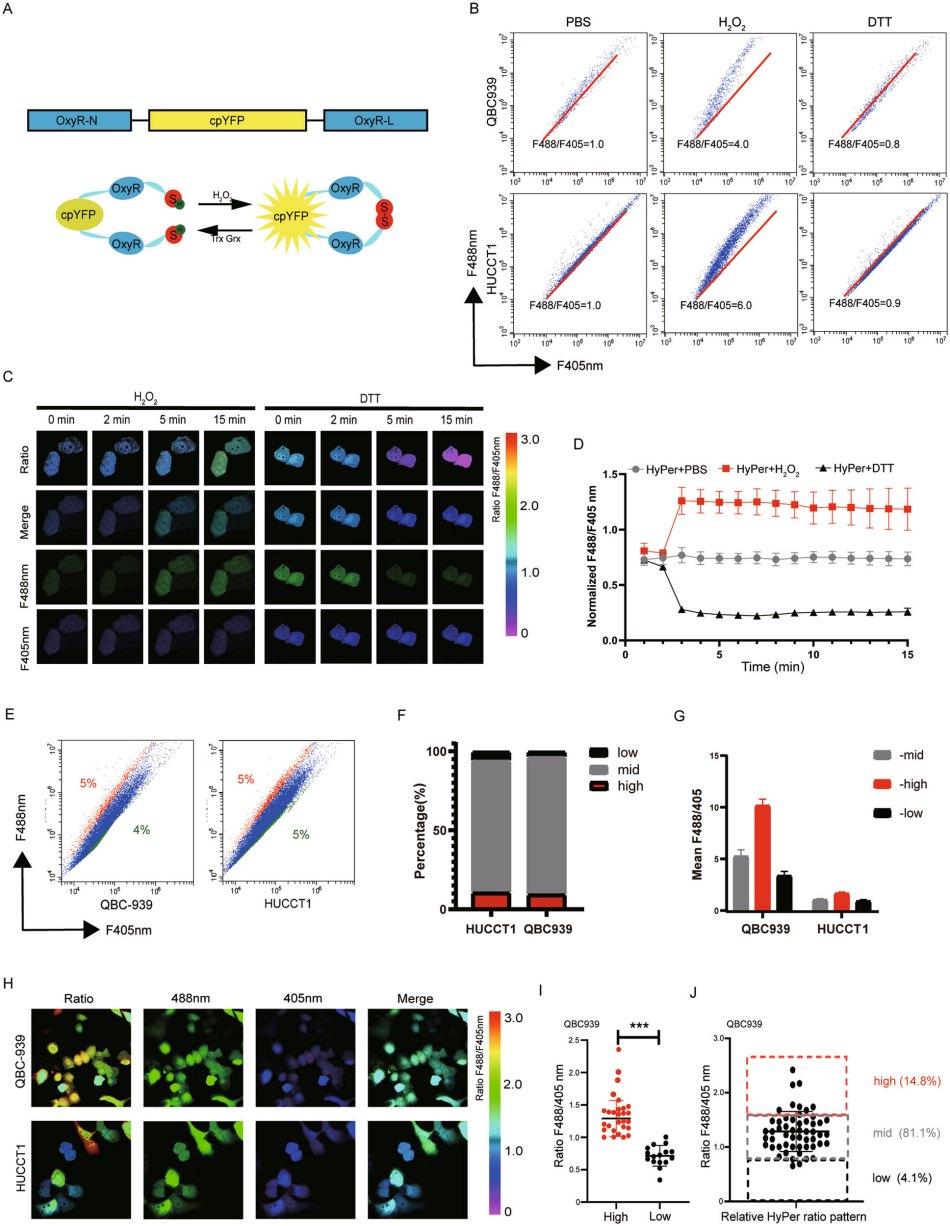
The redox status was heterogeneous in cholangiocarcinoma, which was revealed by HyPer3. **A** The scheme of HyPer3 structure and its oxidation and reduction reaction. **B, C** QBC939 and HUCCT1 cells were transfected with HyPer3 plasmid for 48 h and the ratio of fluorescence (F488/F405nm) was observed under flow-cytometric analysis and confocal microscopy with the treatment of PBS, hydrogen peroxide (100 μM), and DTT (5 mM), respectively. **D** Quantification of **C**. *n* = 10 cells/group. **E** Percentage of ROS differed subpopulations and their accurate ratio of QBC939 and HUCCT1 was measured by flow-cytometric analysis. **F, G** Quantification of **E**. Each experiment was performed in triplicate. **H** Percentage of ROS differed subpopulations and their accurate ratio of QBC939 and HUCCT1 was measured by confocal microscopy. **I**, **J** Quantification of **H**. Each experiment was performed in triplicate. *p < 0.05; **p < 0.01; ***p < 0.001.

The HyPer3 probe is a genetically encoded biosensor that enables real-time imaging of hydrogen peroxide with whole or individual compartments of the cell^22–24^. The probe consists of two OxyR domains with one integrated cpYFP domain. Once hydrogen peroxide reacts with the OxyR domain in the C-terminus, the disulfide bond between Cys199 and Cys208 immediately forms^22,25^ (Fig. 1A). Following disulfide bond formation by hydrogen peroxide, the integrated cpYFP domain undergoes conformational changes and is finally transmitted to the fluorescent protein^22^. Furthermore, the HyPer3 probe also acts as a ratiometric sensor that can vividly reflect the above intramolecular reorganizations. The spectral characteristics of the HyPer3 probe possess two fluorescence excitation peaks (F488 nm and F405 nm) and one fluorescence emission peak (F543 nm). Once under hydrogen peroxide stress, the intensity of the F405 nm excitation peak decreases, yet the F488 nm peak increases.

We first ectopically expressed HyPer3 in 2 kinds of human CCA cell lines, QBC939 and HUCCT1, and then determined the fluorescence ratio when excited at 488 nm and 405 nm when cells were in resting states or upon hydrogen peroxide or DTT treatment by flow cytometric analysis. Exogenous hydrogenous peroxide induced an instantaneous increase in the fluorescence ratios by ~6.0-fold in these two types of CCA cell lines, while exogenous DTT generally led to an inverse decrease in the fluorescence ratios by ~0.8-fold (Fig. 1B).

To further strictly confirm the sensitivity of the HyPer3 probe upon different stimulations, we subjected QBC939 cells to different treatments with hydrogen peroxide and DTT under confocal microscopy. The ratio of the HyPer3 probe fluorescence was markedly increased upon hydrogen peroxide stimulation and slightly decreased when NAC was added (Fig. 1C, D). Above all, we confirmed that the HyPer3 probe, which acts as a genetically encoded hydrogen peroxide biosensor, can dynamically reflect cellular ROS levels and the redox status under flow cytometric analysis or confocal microscopy.

We then examined the ROS level and redox status in the typical CCA cell lines QBC939 and HUCCT1. Following transfection of the HyPer3 probe into QBC939 and HUCCT1 cells, flow cytometric analysis and confocal microscopy tests were performed. Through flow cytometric analysis, we found that CCA cells exhibited a heterogeneous redox status, which was distinguished by the ratio of fluorescence of the HyPer3 probe, and we marked them as HyPer-mid, HyPer-high, and HyPer-low (Fig. 1E). Moreover, the proportions of each subgroup were close (Fig. 1F), while the ratio of the fluorescence of HyPer3 possessed a huge gap in that QBC939 tended to be more characterized among each subgroup (Fig. 1G). The confocal microscopy test showed similar results to the flow cytometric analysis in that the redox status of CCA cells was heterogeneous (Fig. 1H). Due to the larger gap from QBC939 to HUCCT1, we statistically calculated the ratio in QBC939 cells under confocal microscopy, which could be shown as a histogram with three distinct populations, HyPer-mid (81.1% with a mean F488 nm/F405 nm of 0.8~1.7), HyPer-high (14.8% with a mean F488 nm/F405 nm of 0.8~2.8) and HyPer-low (4.1% with a mean F488 nm/F405 nm of 0~0.8) (Fig. 1I, J).

Above all, we found that the redox status of cholangiocarcinoma was heterogeneous and consisted of three distinct populations: HyPer-mid, HyPer-high, and HyPer-low. The subpopulations with different redox statuses can be efficiently sensitively distinguished by the genetically encoded biosensor HyPer3 probe.

### Different subpopulations of the cellular redox status in QBC939 elicited diverse chemoresistance to gemcitabine

**Fig. 2:**
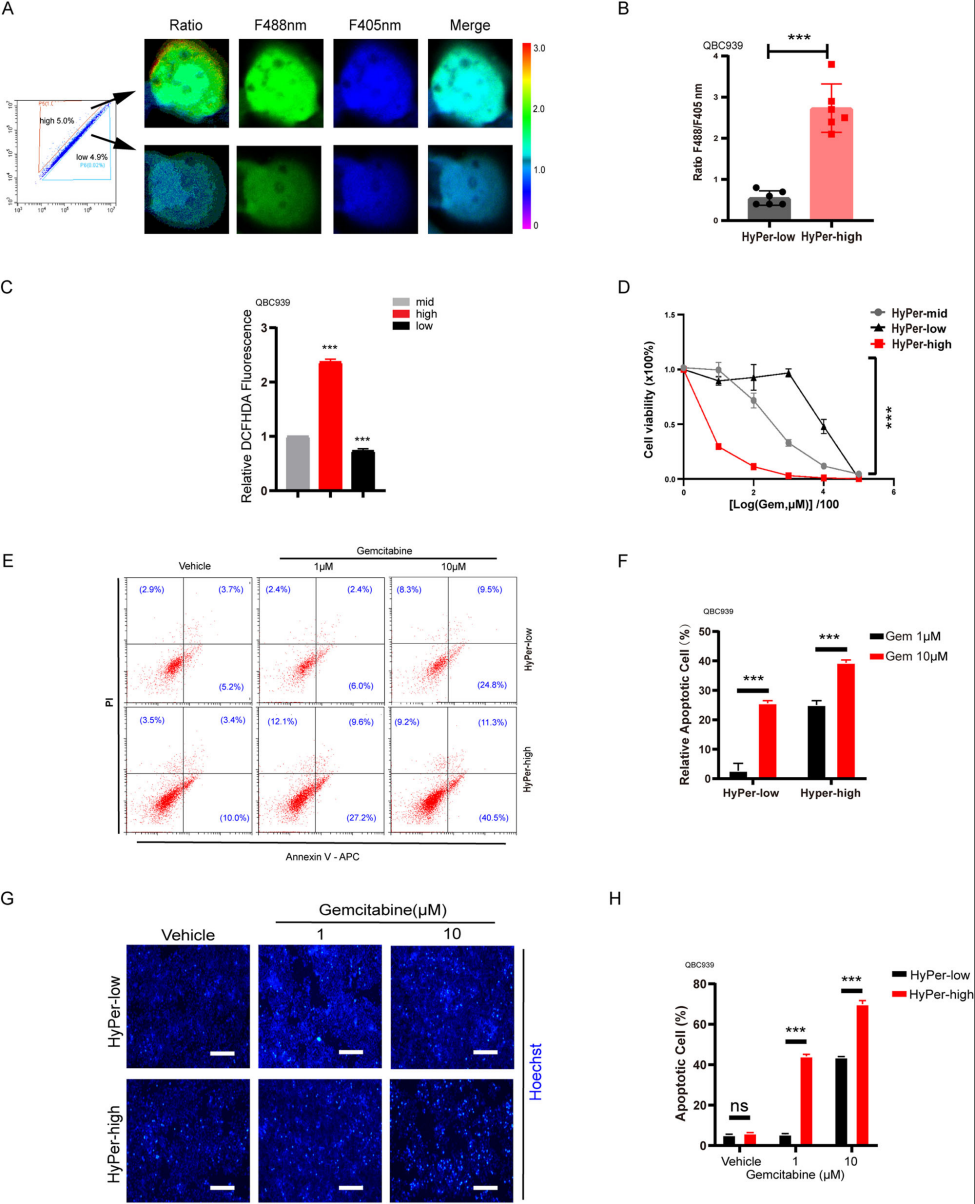
Different subpopulations of the cellular redox status in QBC939 elicited diverse chemoresistance to gemcitabine. **A** QBC939 cells were transfected with the HyPer3 probe plasmid for 48 h and then were sorted by fluorescence-activated cell sorting. Sorted cells were checked by confocal microscopy. **B** Quantification of **A**. **C** Sorted cells were checked by DCFHDA under microplate reader. **D** Sorted cells were treated with Gemcitabine at the indicated concentrations for 48 h. Cell viability was measured using CCK8. **E** The sorted cells were seeding in 6 well plates for 24 h and followed with gemcitabine treatment for 48 h at 1 and 10 μM. Apoptotic assay by Annexin-V/PI staining for flow cytometry after gemcitabine treatment for 48 h. **F** Quantification of **E**. **G** Apoptotic assay by Hoechst dye for immunofluorescence after gemcitabine treatment for 48 h. **H** Quantification of **G**. Each experiment was performed in triplicate. *p < 0.05; **p < 0.01; ***p < 0.001.

Having characterized that CCA cells were heterogeneous in their redox status, we aimed to identify the relationship between the cellular redox status and CCA gemcitabine chemoresistance by sorting the subpopulations with fluorescence-activated cell sorting (FACS) under the guidance of the HyPer3 probe. To control the sorting specificity, we checked the ratio of HyPer3 fluorescence in HyPer-high and HyPer-low cells (Fig. 2A). The sorted populations of QBC939 preserved the ratio gap between the HyPer-high and HyPer-low subpopulations (Fig. 2B). Furthermore we applied DCFHDA to make a further confirmation (Fig. 2C). The correlation was confirmed in the three subpopulations of QBC939, HyPer-mid, HyPer-high, and HyPer-low, by cell-counting kit 8 assays with gradient concentrations (from 0 to 100 μM) of gemcitabine, and HyPer-low CCA cells were characterized by higher resistance to gemcitabine (Fig. 2D). In a second complementary research approach, we used an apoptotic assay to identify the relationship between the redox status and gemcitabine chemoresistance (Fig. 2E, F). By Annexin-V/PI staining for flow cytometric analysis, the HyPer-low subpopulation of CCA cells exhibited a lower apoptosis rate than the HyPer-high subpopulation under gemcitabine treatment from 0 to 10 μM (Fig. 2E, F). Moreover, the apoptotic assay using Hoechst immunofluorescence staining led the same conclusion as the apoptotic assay using Annexin-V/PI staining for flow cytometric analysis, in which the HyPer-low subpopulation of CCA cells possesses a higher rate of gemcitabine chemoresistance (Fig. 2G, H). Taken together, these data indicate that the great chemoresistance of the HyPer-low subpopulation to gemcitabine may be the inner reason for the chemoresistance of CCA to gemcitabine.

### The different subpopulations of cellular redox status in QBC939 elicited diverse chemoresistance to gemcitabine in the xenograft nude mouse model

**Fig. 3:**
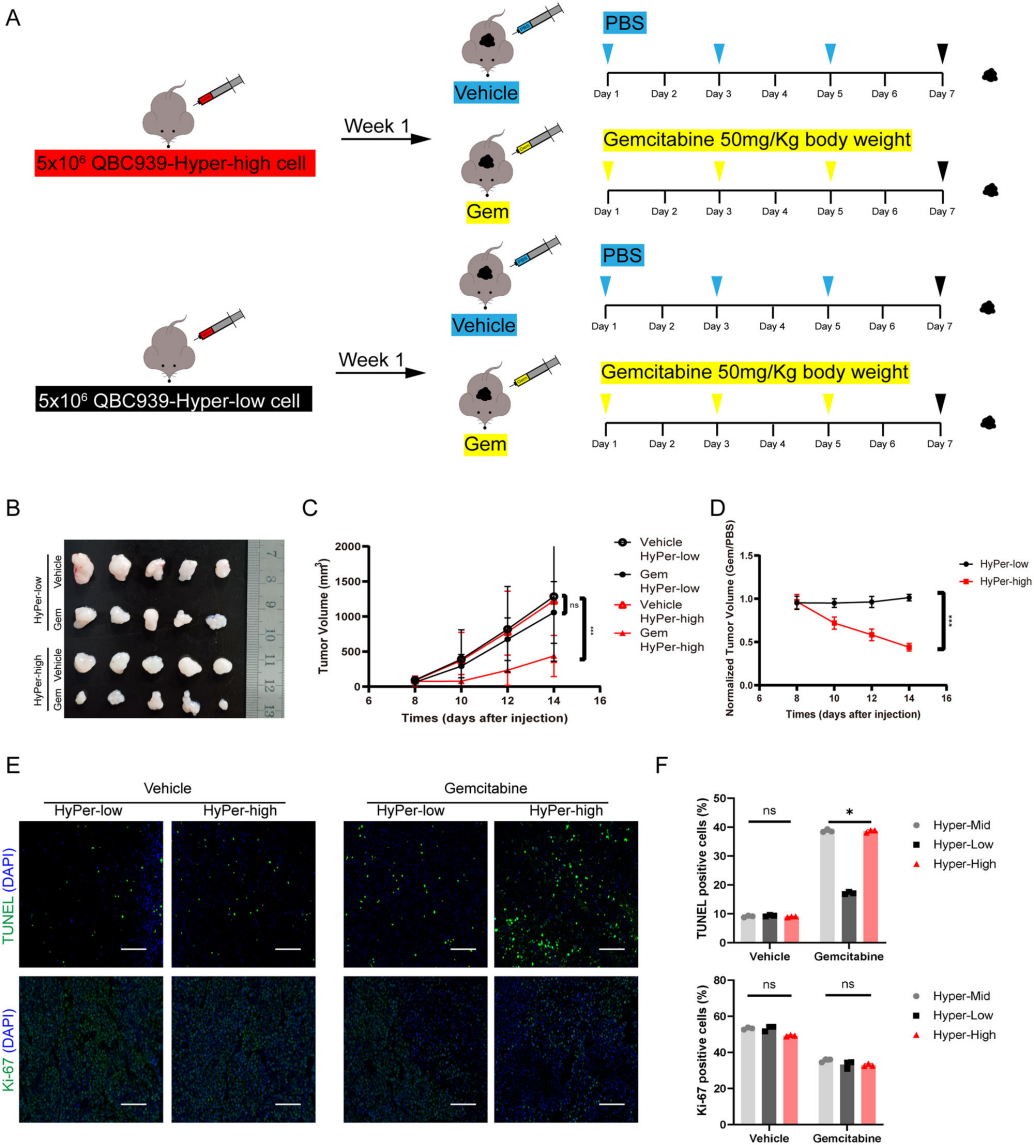
The different subpopulations of cellular redox status in QBC939 elicited diverse chemoresistance to gemcitabine in the xenograft nude mouse model. **A** Schematic representation of the in vivo nude mice model. **B**–**D** Represent xenograft growth inhibition of different subpopulation of CCA cells under the treatment gemcitabine at the indicated concentration via intraperitoneal injection (i.p.) in QBC939 cell xenografts. **E** After treatment with gemcitabine, the TUNEL(up) and Ki-67(down) staining in paraffin-fixed xenograft tissue after scarification was detected using immunofluorescence staining. **F** Quantification of **E**. *p < 0.05; **p < 0.01; ***p < 0.001.

We further established that HyPer-low CCA cells possess higher gemcitabine chemoresistance than other subpopulations in vivo. We used a nude mouse model in which nude mice bearing tumors derived from HyPer-high and HyPer-low CCA cells from QBC939 cells were treated with vehicle (PBS) and gemcitabine (50 mg/kg body weight, intraperitoneal injection) every 2 days, and tumor volumes were measured at the same time (Fig. 3A). When HyPer-high and HyPer-low xenograft nude mice were treated with vehicle or gemcitabine, the HyPer-low subpopulation’s tumor growth was not impaired by the gemcitabine compound but arrested when the Hyper-high subpopulation’s tumor was treated with gemcitabine (Fig. 3B–D). Moreover, we further confirmed the differences in apoptosis and proliferation by immunofluorescence TUNEL and Ki-67 staining, which showed that the HyPer-low subpopulation possessed a lower apoptotic rate than the HyPer-high subpopulation under gemcitabine treatment, while a moderate decrease in proliferation was shared with no difference (Fig. 3E, F). Together, these data are consistent with the correlation between the different cellular redox statuses of CCA cells and gemcitabine chemoresistance, which is extremely severe in the HyPer-low subpopulation of CCA cells.

### *MTHFD1* contributes to different cellular redox statuses and therefore chemoresistance to gemcitabine in cholangiocarcinoma

**Fig. 4:**
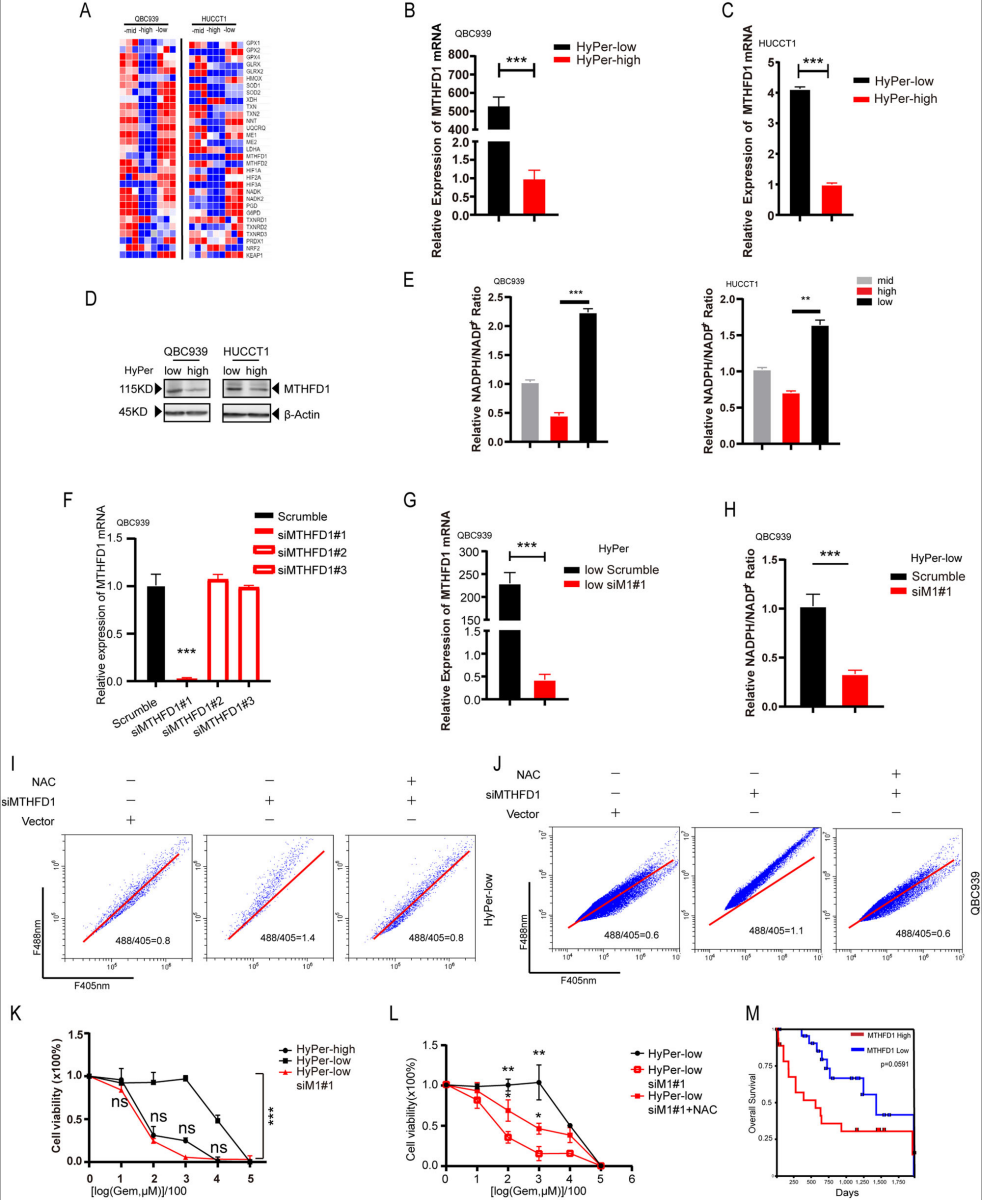
MTHFD1 contributes to different cellular redox statuses and therefore chemoresistance to gemcitabine in cholangiocarcinoma. **A** Heat map of reductase encoded gene in each subpopulation by quantitative real-time PCR. **B**, **C** Relative expression of MTHFD1 mRNA in QBC939 and HUCCT1 respectively by quantitative real-time PCR. **D** HyPer-Low subpopulation of CCA cells possess a higher expression level of MTHFD1 protein. **E** Ratio of cellular NADPH/NADP^+^ in each subpopulation by microplate reader. **F**, **G** MTHFD1 knockdown efficiency by short interfering RNA was detected by quantitative real-time PCR. **H** Ratio of cellular NADPH/NADP^+^ in Scumble-treat and siM1#1 treat HyPer-low CCA cells by microplate reader. **I**, **J** QBC939 and HyPer-Low subpopulation of CCA cells were treated with short interfering RNA of MTHFD1, or its vector or NAC. The ratio of fluorescence was detected by flow-cytometric analysis. **K**, **L** HyPer-High and HyPer-Low subpopulation of CCA cells and siM1#1 HyPer-Low cells were treated with Gemcitabine or NAC at the indicated concentrations for 48 h. Cell viability was measured using CCK8. **M** Quantification of overall survival data from TCGA database. Each experiment was performed in triplicate. *p < 0.05; **p < 0.01; ***p < 0.001.

Based on the higher gemcitabine chemoresistance in the HyPer-low subpopulation, we speculated that a key gene that encodes a reductase or an ROS scavenger may function. To delineate the relationship between cellular redox status and gemcitabine chemoresistance, we screened all of the candidates involved in reducing metabolism by quantitative real-time PCR. Among them, *MTHFD1* was found to have the greatest increase in mRNA in HyPer-low CCA cells compared with HyPer-high CCA cells (Fig. 4A). The data were replicated with a single-gene test by quantitative real-time PCR (Fig. 4B). Moreover, in addition to qPCR, western blot analysis further proved that HyPer-low CCA cells were characterized by higher *MTHFD1* levels (Fig. 4D). To ensure cell line independence, we confirmed that the HyPer-low subpopulation of CCA cells possesses a higher level of *MTHFD1* both in mRNA and protein in HUCCT1 (Fig. 4A, C, D). The evidence of relatively higher *MTHFD1* expression in the HyPer-low subpopulation CCA cells than in the HyPer-high cells combined with the higher gemcitabine chemoresistance in the HyPer-low CCA cells encouraged us to determine whether *MTHFD1* serves as one of the key regulators linking the cellular redox status with gemcitabine chemoresistance. *MTHFD1* is one of the members of C-1-tetrahydrofolate synthases that catalyses the interconversion of tetrahydrofolate (THF), 10-formyl-tetrahydrofolate, 5,10-methyltetrahydrofolate, and 5,10-methylenetetrahydrofolate with NADPH generated as a by-product^26^. Due to that NADPH was generated as a byproduct followed with increased *MTHFD1* expression, cellular NADPH/NADP^+^ ratio was detected in each subpopulation, respectively. At the basal level, HyPer-low CCA cells exhibited a higher NADPH/NADP^+^ ratio while HyPer-high cells go the reverse way which means the increased expression of *MTHFD1* in HyPer-low cells will finally contribute to cellular NADPH increasing (Fig. 4E). To determine the functions of *MTHFD1* in cellular redox regulation and CCA gemcitabine chemoresistance, we silenced *MTHFD1* in the human CCA cell line QBC939 and its HyPer-low subpopulation cells with a short interfering RNA (siRNA)-mediated gene knockdown approach (Fig. 4F), and siMTHFD1#1 (siM1#1) had a marked decrease in the mRNA levels of both QBC939 and its HyPer-low subpopulation cells (Fig. 4G). Meanwhile, with *MTHFD1* knockdown, the ratio of cellular NADPH in HyPer-low cells experienced a dramatic decrease (Fig. 4H). Following *MTHFD1* knockdown, both HyPer3 QBC939 cells and HyPer-low subpopulation cells exhibited a notable increase in the fluorescence ratio, while the antioxidant and free radical scavenger N-acetyl-L-cysteine (NAC) effectively reversed the fluorescence ratio increase (Fig. 4I, J). We next asked whether knockdown of *MTHFD1* altered the gemcitabine chemoresistance in the HyPer-low subpopulation CCA cells. Compared with the HyPer-high subpopulation CCA cells, CCK8 assays indicated that HyPer-low CCA cells with higher expression levels of *MTHFD1* exhibited significant chemoresistance to gemcitabine, whereas *MTHFD1* knockdown transformed the gemcitabine chemoresistance of HyPer-low CCA cells into sensitivity, and this transformation could be reversed by NAC treatment (Fig. 4K, L). With further confirmation, the overall survival rate data from the TCGA database displayed a difference between cholangiocarcinoma patients with higher or lower expression of *MTHFD1* (Fig. 4M). Taken together, we proved that *MTHFD1* overexpression links the cellular redox status with enhanced gemcitabine chemoresistance in the HyPer-low subpopulation CCA cells and cholangiocarcinoma cells.

### Methotrexate synergized with gemcitabine in cholangiocarcinoma via *MTHFD1*-mediated ROS restoration

**Fig. 5:**
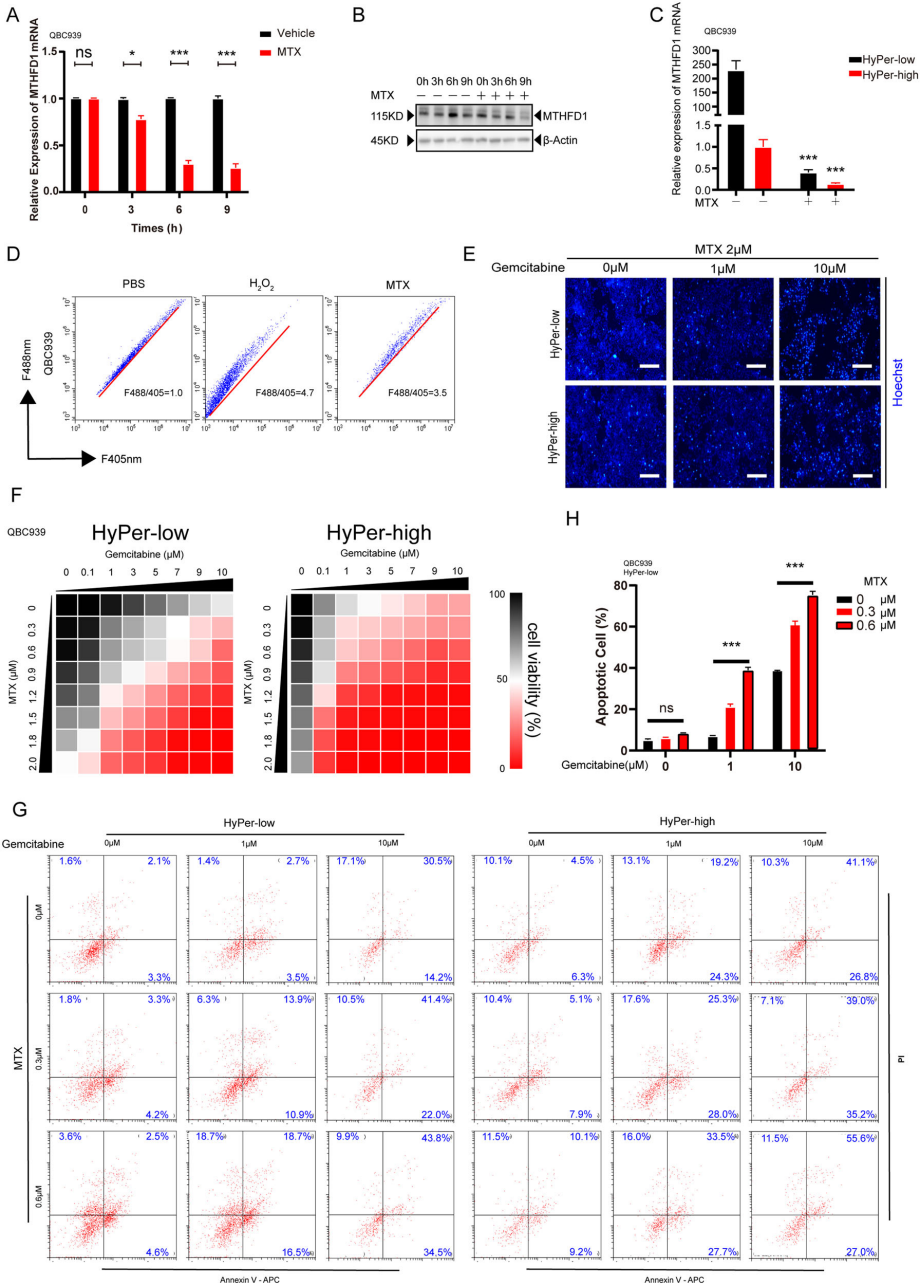
Methotrexate synergized with gemcitabine in cholangiocarcinoma via *MTHFD1*-mediated ROS restoration. **A**, **B** QBC939 was treated with MTX at the indicated concentrations or PBS for 48 h. Relative expression of *MTHFD1* of mRNA and protein was measured. **C** HyPer-Low and HyPer-High subpopulations were treated with MTX at the indicated concentrations for 48 h. Relative expression of *MTHFD1* mRNA was measured. **D** QBC939 was treated with PBS, H_2_O_2_, and MTX respectively at the indicated concentration. The ratio of the HyPer3 probe was measured by flow-cytometric analysis. **E** The sorted cells were seeded in 6 well plates for 24 h and followed with gemcitabine treatment for 48 h at 1 and 10 μM combine with MTX. Apoptotic assay by Hoechst dye for immunofluorescence after gemcitabine treatment for 48 h. **F** Sorted cells were treated with Gemcitabine at the indicated concentrations with MTX at indicated concentrations respectively for 48 h. Cell viability was measured using CCK8. **G** Apoptotic assay by Annexin-V/PI staining for flow cytometry after treated with gemcitabine at the indicated concentrations with MTX at indicated concentrations respectively for 48 h for sorted cells. **H** Quantification of **G**. Each experiment was performed in triplicate.*p < 0.05; **p < 0.01; ***p < 0.001.

Based on the transformation from gemcitabine chemoresistance to sensitivity after *MTHFD1* was knocked down, we hypothesized that antifolates might synergize with gemcitabine to cure patients who are resistant to gemcitabine. Methotrexate (MTX), an antifolate compound, was considered a candidate with great possibility due to its *MTHFD1* inhibiting effect on dihydrofolate reductase (DHFR) and reduced cellular NADPH at the same time. To test this hypothesis, we applied MTX and gemcitabine alone and in combination with QBC939 and its HyPer-low subpopulation CCA cells. Alone with MTX treatment, *MTHFD1* mRNA and its protein exhibited a great decrease in both QBC939 and its HyPer-low subpopulation CCA cells (Fig. 5A–C). We next tested whether the cellular redox status of QBC939 was affected by MTX treatment. Compared with the control treatment of PBS, the fluorescence ratio of the HyPer3 probe by flow cytometric analysis revealed that MTX treatment significantly increased cellular ROS (Fig. 5D). Following the increased cellular ROS, the apoptosis rate was increased at the same time, while the difference in apoptosis between HyPer-low and HyPer-high CCA cells also disappeared by Hoechst immunofluorescence staining (Fig. 5E). For further confirmation, a dose-dependent manner of MTX combine with gemcitabine was adopt. In contrast to the poor response to gemcitabine individually in the HyPer-low subpopulation of CCA cells, the CCK-8 assay proved that the combination of MTX and gemcitabine impaired cell viability in HyPer-low CCA cells under gemcitabine treatment (Fig. 5F) and Annexin-V/PI staining for flow cytometric analysis reassure our conclusion (Fig. 5G, H).

## Discussion

**Fig. 6:**
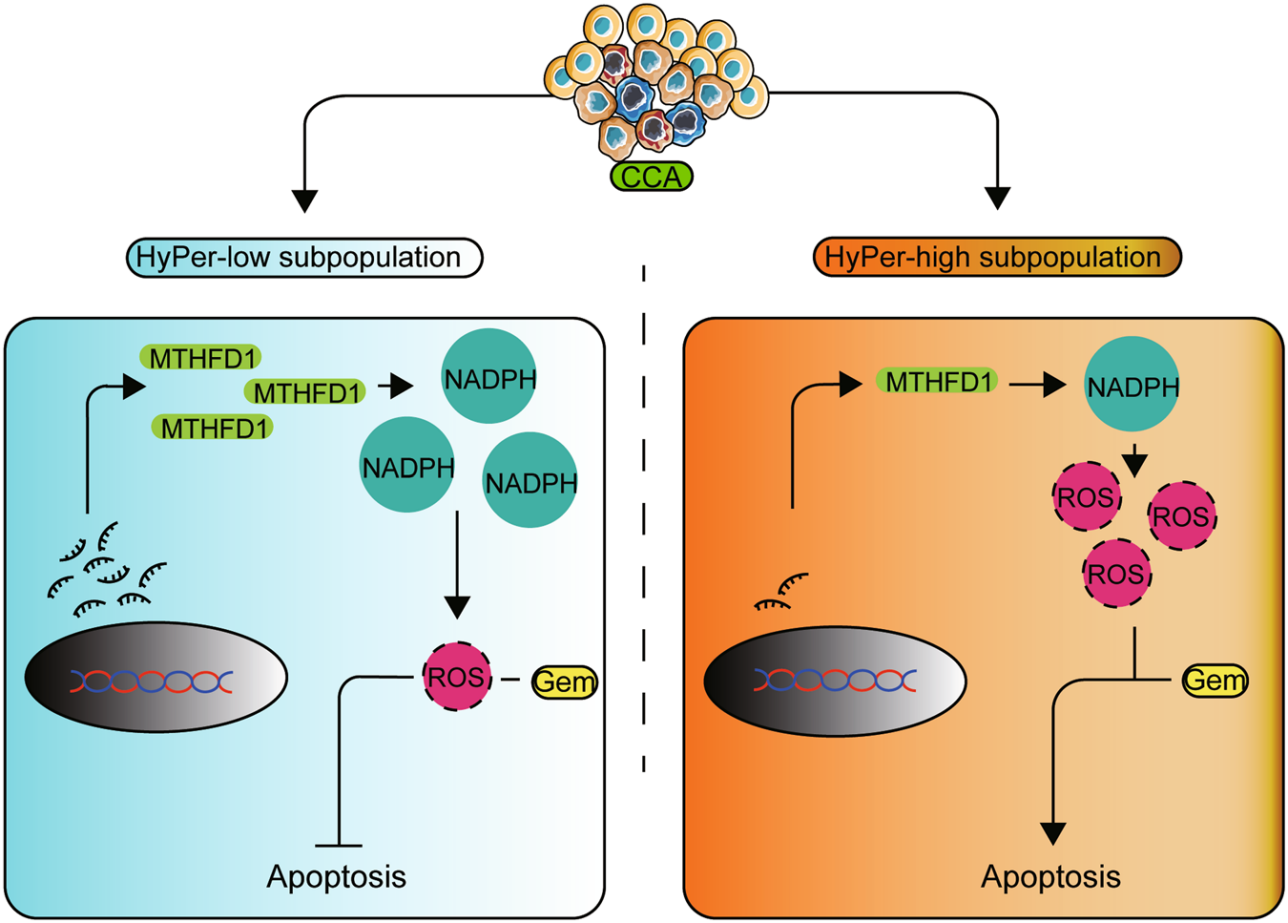
A proposed model of HyPer-Low subpopulation of CCA cells with relative high expression of MTHFD1 impairs Gemcitabine-induced apoptosis in cholangiocarcinoma.

In the present study, working with a novel gene-encoded fluorescence probe that is sensitive to hydrogen peroxide, we sorted the cellular redox statuses of different subpopulations of CCA cells and identified a determinate subpopulation of CCA cells and genes that are essential for gemcitabine chemoresistance in CCA. Our data show that the HyPer-low subpopulation, which is characterized by a lower cellular redox status, is highly associated with gemcitabine chemoresistance. The key link between lower cellular redox status and gemcitabine chemoresistance is the high expression of *MTHFD1* in the HyPer-low subpopulation of CCA cells. Mechanistically, as a key enzyme that catalyses the transversion of tetrahydrofolate (THF), 10-formyl-tetrahydrofolate, 5,10-methyltetrahydrofolate, and 5,10-methylenetetrahydrofolate^26^, NADPH was generated as a by-product from the reduction of NADP, and the catalytic reaction and cellular redox status were changed. As a result of increased cellular NAPDH, gemcitabine-induced apoptosis was efficiently inhibited, which could be restored by *MTHFD1* knockdown. Based on the above research, we provide a potential treatment strategy in which a combination of gemcitabine and the antifolate compound MTX can effectively impair tumor growth in cholangiocarcinoma resulting from restored cellular ROS content. We support the hypothesis that *MTHFD1* overexpression related to a lower cellular redox status leads to gemcitabine chemoresistance in CCA cells, which is highly associated with the much lower cell viability of HyPer-high than HyPer-low CCA cells under gemcitabine treatment, indicating that *MTHFD1* expression is highly correlated with gemcitabine chemoresistance in CCA cells (Fig. 6).

As an antitumour chemotherapeutic due to its role as a cytidine analog, gemcitabine efficiently works with its metabolites dFdCDP and dFdCTP, which can decrease competing deoxyribonucleotide pools and lead to the termination of DNA chain elongation, respectively^27^. In clinical practice over the years, gemcitabine has been broadly used for its capacity to induce apoptosis either single-use in pancreatic cancer or combined-use in non-small cell lung cancer, bladder cancer, breast cancer, etc., with unclear mechanisms^28–32^. In recent decades, an increasing number of studies have confirmed that the cellular redox status or reactive oxygen species (ROS) play an important role in gemcitabine-induced cancer cell apoptosis via direct killing or signal transduction^33,34^. We also confirmed that CCA cells with different cellular ROS exhibited distinct gemcitabine responses. As Huanchen Sha et al. and Yunfeng Zhao et al. reported, ROS-related genes such as *NRF2* and *UCP2* can impair gemcitabine-induced apoptosis by downregulating *NAF-1* or enhancing the stemness of cancer cells^10,35^. Unexpectedly but reasonably, we found that the severe gemcitabine chemoresistance in CCA, especially in the HyPer-low subpopulation of CCA cells, was due to the relatively high expression of *MTHFD1*, the key enzyme in folate metabolism. Under the catalysis of *MTHFD1* during the transversion of the isoforms of THF, NADPH was produced as a by-product and imbalanced the cellular redox status. In the past, the role of *MTHFD1* in folate metabolism was believed to thoroughly influence the recurrence of HCC^21^, neural tube defects^36^, and melanoma metastasis^37^ and induce intestinal carcinogenesis^38^. In the past 5 years, *MTHFD1* was found to regulate hypertension by DNA methylation^39^ and regulate transcription by interacting with *BRD4*^26^. Compared to the above studies, our findings proved that *MTHFD1* is quite important for CCA’s gemcitabine chemoresistance but further found that the correlation between higher expression of *MTHFD1* and gemcitabine chemoresistance resulted in an influence on the cellular redox status due to the overproduction of NADHP under this condition. Once *MTHFD1* was knocked down by short interfering RNA, severe gemcitabine chemoresistance was greatly alleviated, accompanied by the differences between the cellular redox statuses of the HyPer-low and HyPer-high subpopulations being effectively canceled. However, the results can also be complicated because of *MTHFD1*’s enzyme activity. In addition, *MTHFD1* also plays an important role in the stemness of cancer cells, while cancer stem cells are also highly associated with chemoresistance^40^. Our data effectively exhibit the severe gemcitabine chemoresistance in HyPer-low CCA cells with high expression of *MTHFD1* but neglect to verify the potential possibility that HyPer-low CCA cells have equal CCA stem cells, which should be completed in our future study.

Due to the vigorous proliferation of tumor cells, pathways or processes involved were of concern, folate metabolism was greatly highlighted^41–43^ and antifolate compounds such as MTX were developed. Once again, MTX was considered to be effective by inhibiting cell division and nucleotide/deoxynucleotide synthesis^44,45^. In detail, MTX inhibits dihydrofolate reductase leading to depletion of tetrahydrofolate, which is involved in multiple chemical reactions of homocysteine to methionine, where it acts as a proximal methyl donor. Methionine then transformed into S-adenosylmethionine (SAM), which provides methyl groups for RNA, DNA et al. and finally decreased *MTHFD1* expression. Our data suggest a new target for MTX, which could act as a new strategy to cure CCA patients with gemcitabine chemoresistance. Furthermore, the combination of MTX and gemcitabine is highly recommended as a new strategy for CCA patients who are resistant to gemcitabine.
